# Prescription characteristics of Xue-Fu-Zhu-Yu-Tang in pain management: a population-based study using the National Health Insurance Research Database in Taiwan

**DOI:** 10.3389/fphar.2023.1233156

**Published:** 2023-11-21

**Authors:** Chun-En Kuo, Sheng-Feng Hsu, Ching-Chih Chen, Szu-Ying Wu, Yu-Chiang Hung, Chung Y. Hsu, I.-Ju Tsai, Wen-Long Hu

**Affiliations:** ^1^ Department of Chinese Medicine, Kaohsiung Chang Gung Memorial Hospital and School of Traditional Chinese Medicine, Chang Gung University College of Medicine, Kaohsiung, Taiwan; ^2^ School of Chinese Medicine for Post Baccalaureate, I-Shou University, Kaohsiung, Taiwan; ^3^ Department of Leisure and Sports Management, Cheng Shiu University, Kaohsiung, Taiwan; ^4^ Graduate Institute of Acupuncture Science, China Medical University, Taichung, Taiwan; ^5^ Department of Chinese Medicine, China Medical University Hospital, Taipei Branch, Taipei, Taiwan; ^6^ Graduate Institute of Clinical Medical Science, College of Medicine, China Medical University, Taichung, Taiwan; ^7^ Management Office for Health Data, China Medical University Hospital, Taichung, Taiwan; ^8^ College of Medicine, China Medical University, Taichung, Taiwan; ^9^ Kaohsiung Medical University College of Medicine, Kaohsiung, Taiwan; ^10^ Fooyin University College of Nursing, Kaohsiung, Taiwan

**Keywords:** chest pain, myalgia, lumbago, National Health Insurance Research Database, headache

## Abstract

**Objective:** To explore the prevalence and distinctive features of Xue-Fu-Zhu-Yu-Tang (XFZYT) prescriptions by analyzing the National Health Insurance Research Database (NHIRD) to identify the specific medical problems for which XFZYT is prescribed.

**Methods:** This nationwide, population-based, cross-sectional study included 109,073 XFZYT users and 532,848 XFZYT non-users among Chinese herbal product (CHP) users in NHIRD. Chi-squared tests were used to analyze disparities between the XFZYT user and XFZYT non-user cohorts, and the mean age was evaluated using the Wilcoxon rank-sum test. Logistic regression was used to compute the odds ratios (ORs) and 95% confidence intervals (95% CIs).

**Results:** XFZYT was frequently used to treat pain. The top five conditions for which the Taiwanese traditional Chinese medicine (TCM) practitioners would prescribe XFZYT were chest pain; headache; myalgia and myositis; lumbago; and neuralgia, neuritis, and radiculitis.

**Conclusion:** This study represents an inaugural comprehensive survey conducted on the utilization of XFZYT prescriptions among patients with diverse diseases. XFZYT is mostly used to treat pain conditions in Taiwan. Combined with the combination use of other CHPs, XFZYT is used to treat symptoms of the chest and respiratory system, soft tissue conditions, menstruation disorders, and joint and back discomfort. These results suggest that further clinical trials are warranted to verify the effects of XFZYT in pain management.

## 1 Introduction

Traditional Chinese medicine (TCM) has been used in China and several East Asian territories for millennia. Globally, people are seeking natural remedies with improved efficacy and safety. Therefore, TCM is widely accepted as an alternative or adjunct therapeutic approach in several countries (Tsai et al., 2017). Chinese herbal products (CHPs) are of significant importance in TCM in Taiwan (Hung et al., 2016).

Xue-Fu-Zhu-Yu-Tang (XFZYT) is a traditional Chinese medicinal recipe that was mentioned in the “Corrections of Errors in Medical Works (Yi Lin Gai Cuo),” which was published in 1830 CE during the reign of the Qing dynasty of China. XFZYT is comprised of 11 herbs: *Achyranthes bidentata* Blume (Hui Niu Xi), *Angelica sinensis* (Oliv.) Diels, radix (Dang Gui), *Citrus aurantium* L., fructus (Zhi Ke), *Bupleurum chinense* DC., radix (Chai Hu), *Carthamus tinctorius* L., flos (Hong Hua), *Glycyrrhiza uralensis* Fisch., radix and rhizome (Gan Cao), *Ligusticum chuanxiong* Hort., rhizome (Chuan Xiong), *Paeonia lactiflora* Pall., radix (Chi Shao), *Prunus persica* (L.) Stokes, semen (Tao Ren), *Platycodon grandiflorus* (Jacq.) A. DC., radix (Jie Geng), and *Rehmannia glutinosa* (Gaertn.) Libosch., radix (Sheng Di Huang). According to the TCM principle, the administration of XFZYT enhances the circulation of qi and alleviates blood stasis (Yi et al., 2014). Qi stagnation and blood stasis syndromes primarily present as pain. *Ligusticum chuanxiong* Hort., rhizomes (*Chuan Xiong*) and *A. sinensis* (Oliv.) Diels, radix (*Dang Gui*) were also two of the most frequently used herbal medicines for the treatment of qi stagnation and blood stasis syndromes (Gao et al., 2017).

The National Health Insurance (NHI) was implemented in 1995. By the end of 2002, the NHI program covered 98% of the total population residing in Taiwan (Chen et al., 2014). TCM use has been declared eligible for reimbursement by the NHI since 1996. In Taiwan, individuals have the option to choose between Western medicine and TCM as their preferred healthcare approach. Furthermore, they are granted the freedom to directly access primary care clinics or hospitals without the need for reference (Tsai et al., 2017). The National Health Insurance Research Database (NHIRD) is a comprehensive database associated with the NHI program. It includes registration files and original claim data that are used for payment purposes. Consequently, we conducted comprehensive research on a national scale using a population-based approach. Our analysis included an examination of a cohort of one million patients who were randomly selected from NHIRD in Taiwan from 1996 to 2011 (http://nhird.nhri.org.tw/en/index.htm).

The objective of this study was to examine the frequency and attributes of XFZYT prescriptions using NHIRD analysis to ascertain the circumstances under which XFZYT was prescribed. As no national population-based survey of XFZYT prescriptions has been conducted so far, the findings of this study provide significant insights that may contribute to the advancement of pharmacological research and clinical trials.

## 2 Methods

### 2.1 Data sources

Longitudinal data from NHIRD were collected for analysis from 1996 to 2011. The dataset included the medical records of both outpatient and inpatient visits for a randomly chosen sample of one million insured beneficiaries in Taiwan. The NHI program in Taiwan is renowned for its comprehensive coverage, which has reached an impressive rate of 99.99%. Additional details on NHIRD may be obtained from scholarly publications authored by Hsieh et al. (2019). To protect the confidentiality of the data, all datasets were interconnected using distinct encrypted personal identities. Disease diagnoses were classified based on the International Classification of Diseases, Ninth Revision, Clinical Modification (ICD-9-CM). The present work was deemed exempt from review by the Internal Review Board of China Medical University and Hospital (CMUH104-104 REC2-115) owing to the use of de-identified data.

### 2.2 Study subjects and study variables

**FIGURE 1:**
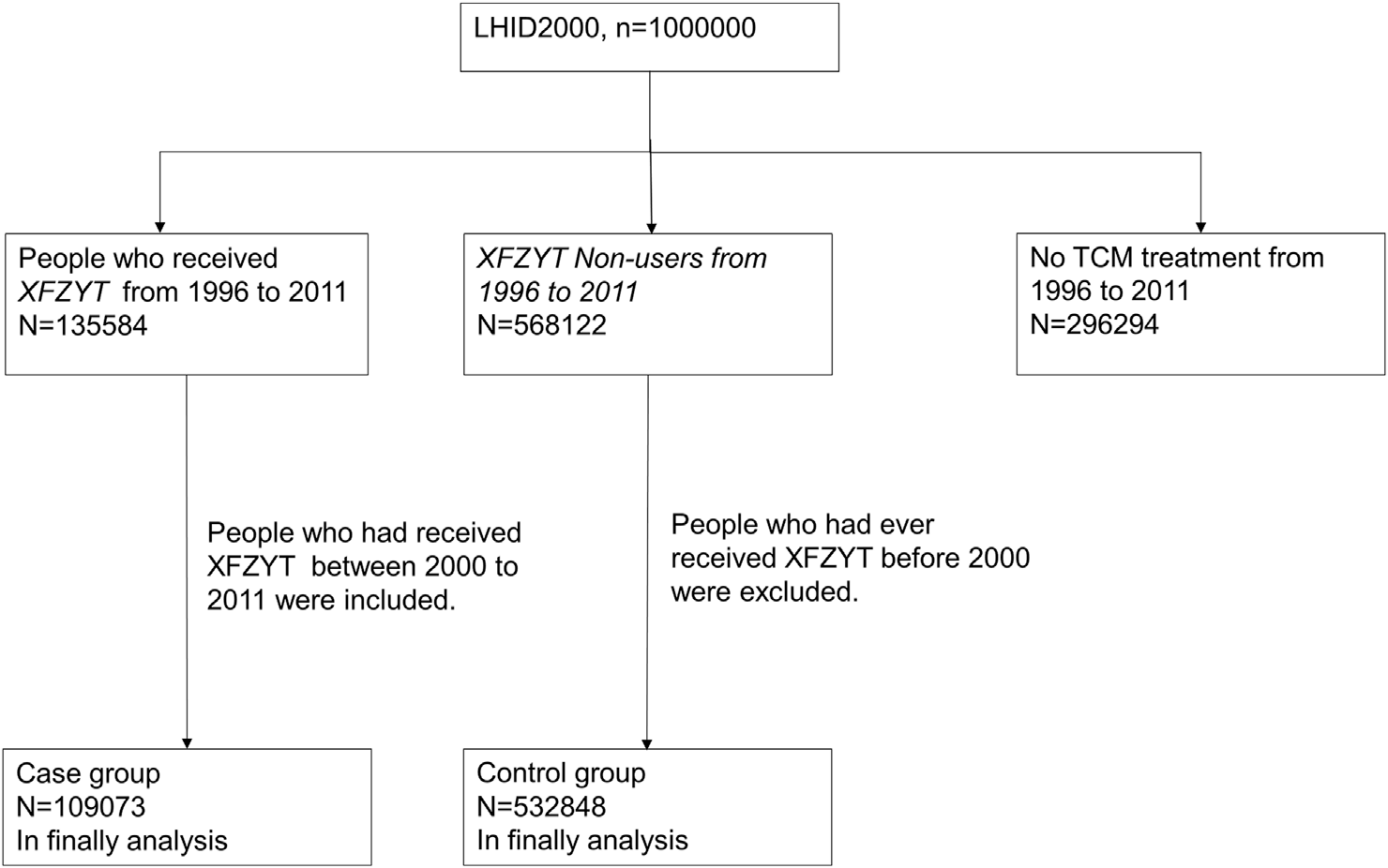
Flow recruitment chart of one million subjects derived from the randomly selected sample obtained from the National Health Insurance Research Database (NHIRD) for the period of 2000–2011 in Taiwan.

Figure 1 depicts the flow recruitment chart of one million participants derived from a randomly selected sample that was extracted from NHIRD in Taiwan, covering the period from 2000 to 2011. Patients (n = 109,073) who were prescribed TCM, including XFZYT, for the first time between 2000 and 2011 were chosen as our case group. The comparison group comprised patients who had been prescribed TCM but did not receive XFZYT (XFZYT non-users). One prescription date was randomly chosen as the index date. The case group comprised 109,073 patients, while the control group included 532,848 patients.

### 2.3 Statistical analysis

Chi-squared tests were used to analyze the differences in features between XFZYT user and XFZYT non-user groups. The mean age was evaluated using the Wilcoxon rank-sum test. Logistic regression was used to compute odds ratios (ORs) along with 95% confidence intervals (95% CIs). Statistical analyses were conducted using SAS version 9.4 (SAS Institute Inc., Cary, NC, USA). Statistical significance was determined to be considerable.

## 3 Results

The mean age of patients who received XFZYT was greater than that of patients who did not receive XFZYT (Table 1; mean age = 40.8 years vs. 36.9 years). The patient population aged between 20 and 64 years comprised the majority of XFZYT users compared with those aged <20 and >65 years. Women seem to use TCM more often than men, and this trend can be observed in both the XFZYT user and XFZYT non-user groups. However, the proportion of female patients in the XFZYT group remained to be greater than that in the control group (57.6% vs. 53.2%). People living in areas with higher urbanization use TCM more than those living in areas with lower urbanization, which is consistent in both groups. There was no difference in occupation distribution between the two groups. Business (OR 1.08, 95% CI 1.06–1.11) and industry (OR 1.08, 95% CI 1.06–1.11) populations tend to use TCM more often than the rest of the occupational populations. In contrast to those whose monthly income exceeds 21,900 NTD, people with a monthly income below this threshold had a higher propensity to use XFZYT. A lower income population was observed among non-users (38.3% vs. 47.1%).

**TABLE 1:** Demographic characteristics and results of CHP XFZYT users from 2000 to 2011 in Taiwan.

Characteristics	User N = 109,073	Non-user N = 532,848	Adjusted OR (95% CI)	*p*-value
Age, years				
<20	12220 (11.2)	116852 (21.9)	1 (reference)	—
20–34	31316 (28.7)	151139 (28.4)	1.90 (1.85, 1.94)	<0.0001
35–49	33925 (31.1)	130730 (24.5)	2.33 (2.27, 2.39)	<0.0001
50–64	20835 (19.1)	81012 (15.2)	2.30 (2.24, 2.36)	<0.0001
65+	10777 (9.9)	53115 (10.0)	1.90 (1.84, 1.95)	<0.0001
Mean (SD)	40.8 (17.1)	36.9 (19.4)	—	<0.0001
Gender				
Women	62825 (57.6)	283563 (53.2)	1.16 (1.14, 1.17)	<0.0001
Men	46248 (42.4)	249285 (46.8)	1 (reference)	—
Urbanization				
1 (highest)	33166 (30.4)	156341 (29.3)	1.05 (1.03, 1.08)	<0.0001
2	33113 (30.4)	156784 (29.4)	1.07 (1.04, 1.09)	<0.0001
3	20273 (18.6)	100672 (18.9)	1.04 (1.02, 1.07)	0.0002
4 (lowest)	22515 (20.6)	119043 (22.3)	1 (reference)	—
Occupation				
Army/Education/Public	11148 (10.2)	54807 (10.3)	1.09 (1.05, 1.12)	<0.0001
Farmer	11721 (10.7)	63553 (11.9)	1 (reference)	—
Fisher	2117 (1.9)	11464 (2.2)	1.03 (0.98, 1.09)	0.2254
Industry	20867 (19.1)	93979 (17.6)	1.12 (1.09, 1.15)	<0.0001
Business	50151 (46.0)	245010 (46.0)	1.08 (1.06, 1.11)	<0.0001
Other	13069 (12.0)	64035 (12.0)	1.11 (1.08 1.14)	<0.0001
Monthly income, NTD				
≤15,840	42334 (38.8)	250942 (47.1)	1 (reference)	—
15,841–21,900	42769 (39.2)	181043 (34.0)	1.09 (1.07, 1.11)	<0.0001
>21,900	23970 (22.0)	100863 (18.9)	1.06 (1.04, 1.08)	<0.0001

**FIGURE 2:**
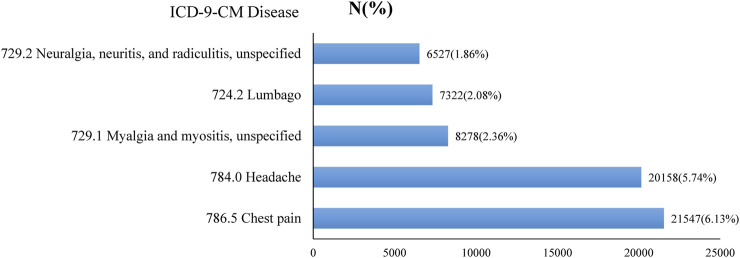
Top five diseases (primary code) treated with CHP XFZYT from 2000 to 2011 in Taiwan.

The top five conditions for which Taiwanese TCM practitioners would prescribe XFZYT were chest pain; headache; myalgia and myositis; lumbago; and neuralgia, neuritis, and radiculitis. All five conditions were related to pain (Figure 2).

Table 2 lists the top seven primary diseases or symptoms frequently treated with XFZYT, either alone or in combination with other herbal formulas, from 2000 to 2011 in Taiwan. Among those, four conditions were directly related to body pain (symptoms affecting the head and neck, other disorders of soft tissues, other and unspecified disorders of back, and contusion of the trunk). The remaining three conditions correlated with pain more or less (symptoms affecting the respiratory system and other chest symptoms, general symptoms, and disorders of menstruation and other abnormal bleeding from the female genital tract), like pain in angina pectoris, chronic pain syndrome, chronic fatigue syndrome, or dysmenorrhea. XFZYT was frequently used for treating symptoms affecting the respiratory system and other chest symptoms (8.43%; ICD-9-CM code: 786) in combination with Chai-Hu-Shu-Gan-Tang and *Curcuma aromatica* Salisb., radix (Yu Jin), general symptoms (7.42%; ICD-9-CM code: 780) in combination with Suan-Zao-Ren-Tang and *Salvia miltiorrhiza* Bunge., radix (Dan Shen), and symptoms affecting the head and neck (5.91%; ICD-9-CM code: 784) in combination with Chuan-Xiong-Cha-Tiao-San and *Pueraria lobata* (Willd.) Ohwi., radix (Ge Gen).

**TABLE 2:** Top seven diseases (primary code) treated with CHP XFZYT from 2000 to 2011 in Taiwan (total 351,377 TCM visits).

ICD-9-CM disease	N (%)	Most frequently combined CHP formula	N (%)	Most frequently combined single CHP	N (%)
786 Symptoms involving the respiratory system and other chest symptoms	29618 (8.43)	Chai-Hu-Shu-Gan-Tang	2344 (0.67)	*Curcuma aromatica* Salisb., radix (Yu Jin)	5744 (1.63)
780 General symptoms	26073 (7.42)	Suan-Zao-Ren-Tang	4500 (1.28)	*Salvia miltiorrhiza* Bunge., radix (Dan Shen)	3627 (1.03)
784 Symptoms involving the head and neck	20783 (5.91)	Chuan-Xiong-Cha-Tiao-San	4983 (1.42)	*Pueraria lobata* (Willd.) Ohwi., radix (Ge Gen)	3593 (1.02)
626 Disorders of menstruation and other abnormal bleeding from the female genital tract	18193 (5.18)	Jia-Wei-Xiao-Yao-San	2967 (0.84)	*Leonurus heterophyllus* Sweet., herba (Yi Mu Cao)	4150 (1.18)
729 Other disorders of soft tissues	15902 (4.53)	Shu-Jing-Huo-Xue-Tang	2360 (0.67)	*Corydalis turtschaninovii* Besser., rhizoma (Yan Hu Suo)	3465 (0.99)
724 Other and unspecified disorders of the back	12133 (3.45)	Du-Huo-Ji-Sheng-Tang	2172 (0.62)	*Corydalis turtschaninovii* Besser., rhizoma (Yan Hu Suo)	2108 (0.6)
922 Contusion of the trunk	10899 (3.1)	Shao-Yao-Gan-Cao-Tang	1480 (0.42)	*Corydalis turtschaninovii* Besser., rhizoma (Yan Hu Suo)	3204 (0.91)

The top 10 most frequent combinations of the two and three diseases for which XFZYT was prescribed, either alone or in combination with other herbal formulas, are shown in Supplementary Tables S1, S2.

## 4 Discussion

XFZYT has long been used in patients with blood stasis and qi stagnation syndromes on the chest and related meridians. However, this research represents an inaugural attempt to use a sample at the national level to elucidate the utilization patterns of XFZYT CHPs in Taiwan, with the aim of discovering further clinical usage of XFZYT. Since NHI has covered over 97% of people in Taiwan since 1997, the possibility of selection bias and recall bias could be excluded. We found that XFZYT was mostly prescribed for symptoms related to pain, including chest pain; headache; myalgia and myositis,; lumbago; and neuralgia, neuritis, and radiculitis (Figure 2). Future studies focusing on pain control using XFZYT hold promise.

The current study revealed that female patients, older age population (but not the oldest population), people residing in an urbanized area, patients with a lower monthly income, and industry or business population were more likely to be prescribed XFZYT. The frequency of XFZYT use was lower among those aged below 20 years, leading to a comparatively higher mean among XFZYT users. These results are not exactly the same as those reported by Hung et al. (2016), in which female patients and the elderly were more likely to be TCM users. Age, sex, and educational level have also been associated with TCM use (Shih et al., 2009). This result may indicate that the groups receiving XFZYT were influenced by sex, age, occupation, and residency. These groups may have a higher tendency toward XFZYT prescriptions by their practitioners because of several possible causes of qi stagnation and blood stasis syndromes, such as body pain, heavy work, stressful life, lack of exercise and outdoor activity, aging, and fluctuation of sex hormones. The elderly population had a higher tendency to have the top five diseases treated with XFZYT: chest pain; headache; myalgia and myositis; lumbago; and neuralgia, neuritis, and radiculitis.

According to the TCM theory, XFZYT was traditionally indicated for qi stagnation and blood stasis and for treating chest pain, chest tightness, headache, insomnia, nausea, and vomiting as presented in “Yi Lin Gai Cuo.” Qi stagnation and blood stasis syndromes were observed when the meridian was blocked in the body, resulting in symptoms such as pain, numbness, and heat or cold. Moreover, according to the TCM theory, the lungs govern qi, the heart governs the blood and blood vessels, and the liver governs the free circulation and storage of the blood. XFZYT contains many herbal medicines that can promote the flow of qi, activate the blood, and supplement the blood simultaneously. In “Yi Lin Gai Cuo,” XFZYT was used to treat blood stagnation above the diaphragm, which resulted in symptoms related to cardiovascular, respiratory, and neuromuscular systems. Compared with our study, most of the diagnoses and symptoms were consistent with ancient XFZYT usage.

XFZYT has been used to treat several medical conditions, including ischemic heart disease (Hung et al., 2015), hyperlipidemia (Chu et al., 2015), platelet aggregation (Huang et al., 2014), migraine (Chang et al., 2014), and prostate cancer (Lin et al., 2012). Lin et al. (2000) showed that XFZYT has properties that are potentially beneficial in the context of hypercholesterolemia, including anti-hypercholesterolemic activity, anti-lipid peroxidation, anti-superoxide anion production, and free radical scavenging activity, as observed in rats. Wang et al. (2006) showed that the intervention resulted in a reduction in endothelium-derived contracting chemicals, including endothelin, and an increase in serum nitric oxide levels in patients diagnosed with unstable angina pectoris. Huang et al. (2014) conducted a pharmacological investigation and showed that XFZYT has anticoagulant and thrombolytic properties. These effects are attributed to its ability to dilate the peripheral blood vessels and coronary arteries, thereby facilitating blood circulation. The analgesic effects of Chai Hu, Chuan Xiong, and Chi Shao have been associated with blood stasis and chest discomfort. Additionally, the reduction in smooth muscle spasms has been associated with the use of Chuan Xiong, Chi Shao, Zhi Shi, and Gan Cao. Liu et al. (2004) showed that XFZYT has the ability to lower the triglyceride levels in the bloodstream, decrease the TXA2/PGI2 ratio, and reduce proinflammatory cytokine synthesis in rats that fed on a diet rich in cholesterol. Zhang et al. (2016) explained that XFZYT has the ability to counteract myocardium fibrosis in hypertensive rats. This is achieved by the enhancement of myocardial cell protection and reduction in TGF-β1 mRNA and protein expression.

The findings of the present study indicate that XFZYT was the medication most often prescribed to patients experiencing chest pain (ICD-9-CM:786.5). XFZYT has been used for the management of cardiovascular diseases for an extensive duration. Hoa et al. (2006) and Chu et al. (2010) reported clinically dependable therapeutic outcomes in the treatment of atherosclerosis and coronary artery disease. XFZYT was shown to mitigate the aberrant metabolic state by modulating many metabolic pathways, resulting in the partial restoration of energy and lipid metabolism dysregulation. XFZYT intervention also exhibited the potential to reduce susceptibility to ketoacidosis, atherosclerosis, and fatty liver disease, which are often associated with high-fat diets. The observed benefits, such as analgesia, may potentially be attributed to the antihyperlipidemic, antioxidant, and anti-inflammatory properties of the constituents of XFZYT (Song et al., 2013), as shown in Table 3.

**TABLE 3:** Traditional use and possible pharmacological effects of the ingredients in XFZYT.

Herb	Traditional use	Possible pharmacological effect
*Angelica sinensis* (Oliv.) Diels, radix (Dang Gui)	Anti-anemic and regulating menstruation pain (Wei et al., 2016)	Anticancer, neuroprotective effect, antioxidant, nephroprotective effects, anti-Alzheimer, hepatoprotective effect, radioprotection, immunoregulation, and anti-inflammatory (Wei et al., 2016)
*Rehmannia glutinosa* Libosch., radix (Sheng Di Huang)	Reducing fever and activating blood circulation, nourishing Yin and tonifying the kidney, and is mainly used for Yin deficiency syndrome in clinics (Zhang et al., 2008)	Used for hemostasis, anti-tumor treatment, immune-enhancement, anti-hypertensive, and anti-diabetics (Zhang et al., 2008)
*Prunus persica* (L.) Stokes, semen (Tao Ren)	Quicken the blood and transform stasis (Xu, 2015)	Anticoagulant, antithrombotic, liver fibrosis prevention, and immunity enhancement (Xu, 2015)
*Carthamus tinctorius* L., flos (Hong Hua)	Activating blood circulation and removing the stasis (Yao et al., 2016)	Antiplatelet aggregation, anticoagulant, antioxidant, and ovarian granulosa cell proliferation (Yao et al., 2016)
*Citrus aurantium* L., fructus (Zhi Ke)	Regulating Qi and promoting gastrointestinal motility (Singh and Mahajan, 2013)	Regulating gastrointestinal motility, lowering blood lipid, and resisting tumor and thrombus (Singh and Mahajan, 2013)
*Paeonia lactiflora* Pall., radix (Chi Shao)	Cooling blood, dissipating blood stasis, and relieving pain (Lu, 2015)	Hepatoprotective effect, anti-tumor, neuroprotection, and cardio-protective effect (Lu, 2015)
*Bupleurum chinense* DC., radix (Chai Hu)	Resolving the exterior, abating heat, coursing the liver and resolving depression, upbearing Yang, and raising the fall (Li et al., 2015)	Anti-tumor, hepatoprotective effect, potential estrogen-like effect, and nephropathy protection (Li et al., 2015)
*Glycyrrhiza uralensis* Fisch., radix and rhizome (Gan Cao)	Supplement and boost central qi, relax tension, and relieve pain (Zhang, 2014)	Anti-arrhythmic, anti-tumor, anti-viral, anti-bacterial, anti-inflammatory, and immune-modulation (Zhang, 2014)
*Platycodon grandiflorus (*Jacq.) A. DC., radix (Jie Geng)	Diffusing the lung and dispersing cold, expelling phlegm, and disinhibiting the throat (Lee et al., 2004)	Relieving cough, reducing sputum, anti-tumor, anti-oxidant, and immune enhancement (Lee et al., 2004)
*Achyranthes bidentata* Blume., radix (Hui Niu Xi)	Supplement the liver and kidney, strengthen sinew and bone, quicken the blood and transform stasis, disinhibit urine and relieve strangury, and conduct blood downward (Liu et al., 2022)	Direct vasodilation and significant effect against the α receptor (Liu et al., 2022)
*Ligusticum chuanxiong* Hort., rhizome (Chuan Xiong)	Quicken the blood, move qi, and relieve pain (Ran et al., 2011)	Antioxidant, neuroprotective, anti-inflammatory, and anti-nociceptive (Ran et al., 2011)

In Taiwan, general symptoms (ICD-9-CM:780) were the second most prevalent diagnosis among patients receiving XFZYT. Sleep disturbance (ICD-9-CM:780.9) has emerged as the prevailing diagnosis among XFZYT users experiencing general symptoms. In clinical practice, the general symptoms include non-specific pain syndrome and fatigue. This population is quite likely to have unsatisfactory sleep quality at the same time. The contemporary approaches of TCM toward chronic fatigue syndrome mostly revolve around the modulation of immunological dysfunction, regulation of an aberrant activity within the hypothalamic–pituitary–adrenal (HPA) axis, and provision of antioxidant properties (Chen et al., 2010). Shao et al. (2020) showed evidence supporting the effectiveness and safety of XFZYT in alleviating symptoms associated with post-stroke depression and improving sleep quality, which was consistent with the observation that XFZYT was used to treat insomnia as presented in “Yi Lin Gai Cuo.”

In Taiwan, symptoms related to the head and neck (ICD-9-CM:784) were the third most prevalent diagnosis among patients receiving XFZYT treatment, and the most commonly associated diagnosis was headache (ICD-9-CM:784.0). Wang et al. (2009) performed a study comprising 70 cases and observed that XFZYT had a good impact on post-stroke headaches. Li and Tan (2015) conducted a meta-analysis and showed that modified XFZYT decoction had a significant effect on the overall cure rate of headache compared to modern medicine.

In our statistics, XFZYT is prescribed in combination with other CHPs to treat symptoms of the chest and respiratory system, soft tissue conditions, menstruation disorders, and joint and back discomfort. The findings of our study are consistent with those of a prior investigation of the possible pharmacological effects of XFZYT. Recent studies on XFZYT have primarily evaluated its cardiovascular effects, lipid-lowering profiles, and neuroprotective effects. A systematic review revealed that XFZYT improved blood pressure symptoms, blood lipid levels, hemorheology, carotid intima-media thickness, and left ventricular mass index (Wang et al., 2015). Therefore, we assure that the application of XFZYT in cardiovascular and chest conditions is widespread among TCM practitioners. However, in this study, we found the massive use of XFZYT in pain management. Using keywords, “XFZYT” and “pain,” we were able to obtain only a few papers and none were really related to primary pain conditions (one mentioned about relieving pain in angina pectoris patients). Based on the examination of this novel aspect, it is suggested that more investigations should be conducted to explore the efficacy of XFZYT in pain management, particularly in the context of chest pain; headache; myalgia and myositis; lumbago; and neuralgia, neuritis, and radiculitis. XFZYT could be considered a prescription for managing diverse pain conditions.

The present study has several limitations. First, this study did not demonstrate the effectiveness of XFZYT. Although NHIRD is a large database, we were unable to obtain prescription data or detailed patient records. Therefore, it was not possible to evaluate the results of using XFZYT. Second, the ICD-9-CM diagnosis, which is used in clinical practice in Western medicine, does not completely reflect TCM diagnosis. However, the NHI database only recognizes ICD-9-CM as the approved coding method. Hence, the establishment of a coding system is of paramount importance in TCM research. Third, the objective of this study was to distinguish between people who had previously taken XFZYT and those who had never used it. To do this, we set the time period 1996–1999 as the washout period. During this period, individuals who did not use XFZYT were classified as non-users. Nevertheless, it is imperative to recognize that this methodology does not provide absolute certainty regarding the absence of prior use of XFZYT among these individuals before 1996, which is a constraint of our research. Fourth, in clinical practice, TCM prescriptions usually consist of multiple CHP combinations, instead of a single formula. This makes it difficult to clarify the relationship between CHP system and diagnosis. However, this study still provides valuable information regarding the application of XFZYT. 

## 5 Conclusion

This study is the first comprehensive investigation for the use of XFZYT prescriptions across a diverse range of medical conditions among patients. We found that XFZYT was mostly applied in conditions relating to pain in Taiwan. The top five conditions for which Taiwanese TCM practitioners would prescribe XFZYT were chest pain; headache; myalgia and myositis; lumbago; and neuralgia, neuritis, and radiculitis. In combination with other CHPs, XFZYT is used to treat the symptoms of the chest and respiratory system, soft tissue conditions, menstruation disorders, and joint and back discomfort. These results suggest that further clinical studies are warranted to validate the therapeutic benefits of XFZYT for pain management.

## Data Availability

The original contributions presented in the study are included in the article/Supplementary Material; further inquiries can be directed to the corresponding author.

## References

[B1] Chang Y. Y. Tsai Y. T. Lai J. N. Yeh C. H. Lin S. K. (2014). The traditional Chinese medicine prescription patterns for migraine patients in Taiwan: a population-based study. J. Ethnopharmacol. 151, 1209–1217. 10.1016/j.jep.2013.12.040 24389028

[B2] Chen F. P. Chang C. M. Hwang S. J. Chen Y. C. Chen F. J. (2014). Chinese herbal prescriptions for osteoarthritis in Taiwan: analysis of national health insurance dataset. BMC Complementary Altern. Med. 14, 91–98. 10.1186/1472-6882-14-91 PMC3973832 24606767

[B3] Chen R. Moriya J. Yamakawa J. Takahashi T. Kanda T. (2010). Traditional Chinese medicine for chronic fatigue syndrome. Evidence-Based Complementary Altern. Med. 7, 3–10. 10.1093/ecam/nen017 PMC2816380 18955323

[B4] Chu F. Y. Wang J. Yao K. W. Li Z. Z. (2010). Effect of Xuefu Zhuyu Capsule on the symptoms and signs and health-related quality of life in the unstable angina patients with blood-stasis syndrome after percutaneous coronary intervention: a randomized controlled trial. Chin. J. Integr. Med. 16, 399–405. 10.1007/s11655-010-9999-9 20535581

[B5] Chu S. M. Shih W. T. Yang Y. H. Chen P. C. Chu Y. H. (2015). Use of traditional Chinese medicine in patients with hyperlipidemia: a population-based study in Taiwan. J. Ethnopharmacol. 168, 129–135. 10.1016/j.jep.2015.03.047 25828254

[B6] Gao J. L. Chen G. He Q. Y. Li J. Wang J. (2017). Analysis of Chinese patent medicine prescriptions for qi stagnation and blood stasis syndrome. Zhongguo Zhong yao za zhi= Zhongguo Zhongyao Zazhi= China J. Chin. Materia Medica 42, 187–191. 10.19540/j.cnki.cjcmm.20161222.005 28945047

[B7] Hoa B. Cao W. D. Yang T. (2006). Intervention of xuefu zhuyu oral liquid on expression of adhesion molecule CDllb/CD18 in neutrophils in patients with ateriosclerosis obliterans. Zhongguo Zhong xi yi jie he za zhi= Chin. J. Integr. Traditional West. Med. 26, 125–127. 16548352

[B8] Hsieh C. Y. Su C. C. Shao S. C. Sung S. F. Lin S. J. Kao Yang Y. H. (2019). Taiwan's national health insurance research database: past and future. Clin. Epidemiol. 11, 349–358. 10.2147/CLEP.S196293 31118821 PMC6509937

[B9] Huang Y. Jiang W. Xiao Y. Wang Y. Wang Y. (2014). Multiobjective optimization on antiplatelet effects of three components combination by quantitative composition-activity relationship modeling and weighted-sum method. Chem. Biol. Drug Des. 84, 513–521. 10.1111/cbdd.12338 24725674

[B10] Hung Y. C. Cheng Y. C. Muo C. H. Chiu H. E. Liu C. T. Hu W. L. (2016). Adjuvant Chinese herbal products for preventing ischemic stroke in patients with atrial fibrillation. Plos one 11, e0159333. 10.1371/journal.pone.0159333 27428543 PMC4948896

[B11] Hung Y. C. Tseng Y. J. Hu W. L. Chen H. J. Li T. C. Tsai P. Y. (2015). Demographic and prescribing patterns of Chinese herbal products for individualized Therapy for ischemic heart disease in taiwan: population-based study. PloS one 10, e0137058. 10.1371/journal.pone.0137058 26322893 PMC4556444

[B12] Lee J. Y. Hwang W. L. Lim S. T. (2004). Antioxidant and anticancer activities of organic extracts from Platycodon grandiflorum A. De Candolle roots. J. Ethnopharmacol. 93, 409–415. 10.1016/j.jep.2004.04.017 15234786

[B13] Li M. F. Tan W. L. (2015). Treatment of migraine with xuefu zhuyu decoction: a meta-analysis. Chin. J. Ethnomedicine Ethopharmacy 24, 29–31.

[B14] Li X. Y. Dou L. W. Sun J. H. Sun R. (2015). Research development on pharmacological effects and toxicity of saikosaponin-d. Chin. J. Pharmacovigil. 12, 207.

[B15] Lin C. C. Yen F. L. Hsu F. F. Lin J. M. (2000). Anti-hypercholesterolaemia, antioxidant activity and free radical scavenger effects of traditional Chinese medicine prescriptions used for stroke. J. Pharm. Pharmacol. 52, 1387–1393. 10.1211/0022357001777388 11186247

[B16] Lin Y. H. Chen K. K. Chiu J. H. (2012). Coprescription of Chinese herbal medicine and western medications among prostate cancer patients: a population-based study in taiwan. Evid. Based Complement. Altern. Med. 2012, 147015. 10.1155/2012/147015 PMC3139871 21792368

[B17] Liu H. X. Mohammed S. A. Lu F. Chen P. Wang Y. Liu S. (2022). Network pharmacology and molecular docking-based mechanism study to reveal antihypertensive effect of gedan jiangya decoction. BioMed Res. Int. 2022, 3353464. 10.1155/2022/3353464 36046450 PMC9423997

[B18] Liu L. Cheng Y. Zhang H. (2004). Phytochemical analysis of anti-atherogenic constituents of Xue-Fu-Zhu-Yu-Tang using HPLC-DAD-ESI-MS. Chem. Pharm. Bull. 52, 1295–1301. 10.1248/cpb.52.1295 15516749

[B19] Lu X. H. (2015). Research progress on chemical constituents of Paeoniae Rubra Radix and their pharmacological effects. Chin. Traditional Herb. Drugs, 595–602.

[B20] Ran X. Ma L. Peng C. Zhang H. Qin L.-P. (2011). Ligusticum chuanxiong Hort: a review of chemistry and pharmacology. Pharm. Biol. 49, 1180–1189. 10.3109/13880209.2011.576346 22014266

[B21] Shao J. Y. Zhou L. M. Shao T. Y. Ding M. R. Jin Z. Q. (2020). Effectiveness and safety of the Xuefu Zhuyu Tang for post-stroke depression: a systematic review and meta-analysis. Eur. J. Integr. Med. 37, 101150. 10.1016/j.eujim.2020.101150

[B22] Shih C. C. Lin J. G. Liao C. C. Su Y. C. (2009). The utilization of traditional Chinese medicine and associated factors in Taiwan in 2002. Chin. Med. J. 122, 1544–1548. 19719945

[B23] Singh I. P. Mahajan S. (2013). Berberine and its derivatives: a patent review (2009–2012). Expert Opin. Ther. Pat. 23, 215–231. 10.1517/13543776.2013.746314 23231038

[B24] Song X. F. Wang J. S. Wang P. G. Tian N. Yang M. H. Kong L. Y. (2013). ¹H NMR-based metabolomics approach to evaluate the effect of Xue-Fu-Zhu-Yu decoction on hyperlipidemia rats induced by high-fat diet. J. Pharm. Biomed. Anal. 78, 202–210. 10.1016/j.jpba.2013.02.014 23501440

[B25] Tsai M. Y. Hu W. L. Lin C. C. Lee Y. C. Chen S. Y. Hung Y. C. (2017). Prescription pattern of Chinese herbal products for heart failure in Taiwan: a population-based study. Int. J. Cardiol. 228, 90–96. 10.1016/j.ijcard.2016.11.172 27863367

[B26] Wang B. X. Dong X. M. Guo A. M. Zhang J. (2006). Effects of xuefu zhuyu decoction on functions of vascular endothelium in patients with unstable angina pectoris. Zhong Xi Yi Jie He Xue Bao 4, 256–259. 10.3736/jcim20060307 16696911

[B27] Wang P. Q. Xiong X. J. Li S. J. (2015). Efficacy and safety of a traditional Chinese herbal formula Xuefu Zhuyu decoction for hypertension: a systematic review and meta-analysis. Medicine 94, e1850. 10.1097/MD.0000000000001850 26496333 PMC4620751

[B28] Wang Y. C. Guo C. H. Chen X. L. (2009). Observation of the curative effect of Xuefu Zhuyu decoction with Baizhi and Huangqi on headache after stroke. Mod. J. Integr. traditional Chin. West. Med. (in Chinese) 18, 2628–2629.

[B29] Wei W. L. Zeng R. Gu C. M. Qu Y. Huang L. F. (2016). Angelica sinensis in China-A review of botanical profile, ethnopharmacology, phytochemistry and chemical analysis. J. Ethnopharmacol. 190, 116–141. 10.1016/j.jep.2016.05.023 27211015

[B30] Xu X. H. (2015). Research progress in persicae semen. Chinese Traditional and Herbal Drugs, 2649–2655.

[B31] Yao D. Wang Z. Miao L. Wang L. Y. (2016). Effects of extracts and isolated compounds from safflower on some index of promoting blood circulation and regulating menstruation. J. Ethnopharmacol. 191, 264–272. 10.1016/j.jep.2016.06.009 27286914

[B32] Yi G. Z. Qiu Y. Q. Xiao Y. Yuan L. X. (2014). The usefulness of xuefu zhuyu tang for patients with angina pectoris: a meta-analysis and systematic review. Evidence-based complementary and alternative medicine 2014, 521602. 10.1155/2014/521602 25254054 PMC4164128

[B33] Zhang G. Yang G. Deng Y. Zhao X. Yang Y. Rao J. (2016). Ameliorative effects of Xue-Fu-Zhu-Yu decoction, Tian-Ma-Gou-Teng-Yin and Wen-Dan decoction on myocardial fibrosis in a hypertensive rat mode. BMC Complement. Altern. Med. 16, 56. 10.1186/s12906-016-1030-3 26852136 PMC4744408

[B34] Zhang L. (2014). A review on pharmacological effects of licorice. Clin J Chin Med 6, 147–149.

[B35] Zhang R. X. Li M. X. Jia Z. P. (2008). Rehmannia glutinosa: review of botany, chemistry and pharmacology. J. Ethnopharmacol. 117, 199–214. 10.1016/j.jep.2008.02.018 18407446

