# Supplementary material for: Prescription characteristics of Xue-Fu-Zhu-Yu-Tang in pain management: a population-based study using the National Health Insurance Research Database in Taiwan
Source: Front Pharmacol. 2023 Nov 21;14:1233156. doi: 10.3389/fphar.2023.1233156 (PMC10703182; doi:10.3389/fphar.2023.1233156)
Supplement: Supplementary file 1 [file Table1.DOCX]

| **Supplementary Table 1. Top 10 two-combined diseases treated with CHP XFZYT from 2000 to 2011 in Taiwan (Total 351377 TCM visits)** | | | | | | |
| --- | --- | --- | --- | --- | --- | --- |
| ICD-9-CM Disease | ICD-9-CM Disease | N (%) | Most frequent combined formula CHP | N (%) | Most frequent combined single CHP | N (%) |
| 729 Other disorders of soft tissues | 780 General symptoms | 331 (0.6) | Suan-zao-ren-tang | 57 (0.02) | *Corydalis turtschaninovii* Besser., rhizoma (*Yan Hu Suo*) | 57 (0.02) |
| 401 Essential hypertension | 780 General symptoms | 300 (0.54) | Tian-ma-gou-teng-yin | 53 (0.02) | *Salvia miltiorrhiza* Bunge., radix (*Dan Shen*) | 85 (0.02) |
| 780 General symptoms | 564 Functional digestive disorders, not elsewhere classified | 288 (0.52) | Xiao-yao-san | 72 (0.02) | *Ziziphus jujuba* Mill., semen (*Suan Zao Ren*) | 39 (0.01) |
| 786 Symptoms involving respiratory system and other chest symptoms | 786 Symptoms involving respiratory system and other chest symptoms | 281 (0.51) | Fu-fang-dan-shen-pian | 101 (0.03) | *Corydalis turtschaninovii* Besser., rhizoma (*Yan Hu Suo*) | 91 (0.03) |
| 626 Disorders of menstruation and other abnormal bleeding from female genital tract | 564 Functional digestive disorders, not elsewhere classified | 272 (0.49) | Jia-wei-xiao-yao-san  Jia-wei-xiao-yao-san | 50 (0.01) | *Rheum officinale* Baill., radix and rhizome (*Da Huang*) | 46 (0.01) |
| 786 Symptoms involving respiratory system and other chest symptoms | 780 General symptoms | 259 (0.47) | Chai-hu-shu-gan-tang | 32 (0.01) | *Ziziphus jujuba* Mill., semen (*Suan Zao Ren*) | 43 (0.01) |
| 784 Symptoms involving head and neck | 780 General symptoms | 257 (0.47) | Jia-wei-xiao-yao-san | 47 (0.01) | *Vitex trifolia* L., semen (*Man Jing Zi*) | 30 (0.01) |
| 626 Disorders of menstruation and other abnormal bleeding from female genital tract | 780 General symptoms | 253 (0.46) | Jia-wei-xiao-yao-san | 49 (0.01) | *Leonurus heterophyllus* Sweet., herba (*Yi Mu Cao*) | 68 (0.02) |
| 250 Diabetes mellitus | 272 Disorders of lipoid metabolism | 228 (0.41) | Zhi-bai-di-huang-wan | 39 (0.01) | *Salvia miltiorrhiza* Bunge., radix (*Dan Shen*) | 69 (0.02) |
| 724 Other and unspecified disorders of back | 729 Other disorders of soft tissues | 227 (0.41) | Shao-yao-gan-cao-tang | 94 (0.03) | *Eucommia ulmoides* Oliv., cortex (*Du Zhong*) | 87 (0.02) |

| **Supplementary Table 2. Top 10 three-combined diseases treated with CHP XFZYT from 2000 to 2011 in Taiwan (Total 351377 TCM visits)** | | | | | | | |
| --- | --- | --- | --- | --- | --- | --- | --- |
| ICD-9-CM Disease | ICD-9-CM Disease | ICD-9-CM Disease | N (%) | Most frequent combined formula CHP | N (%) | Most frequent combined single CHP | N (%) |
| 719 Other and unspecified disorders of joint | 287 Purpura and other hemorrhagic conditions | 287 Purpura and other hemorrhagic conditions | 61 (0.57) | Qiang-Huo-Sheng-Shi-Tang | 26 (0.01) | *Aconitum carmichaelii* Debeaux., radix (*Chuan Wu*) | 3 (0.00) |
| 290 Dementias | 401 Essential hypertension | 272 Disorders of lipoid metabolism | 60 (0.56) | Ban-Xia-Bai-Zhu-Tian-Ma-Tang | 60 (0.02) | *Uncaria rhynchophylla* (Miq.) Miq. ex Havil., caulis(*Gou Teng*) | 60 (0.02) |
| 564 Functional digestive disorders, not elsewhere classified | 564 Functional digestive disorders, not elsewhere classified | 278 Overweight, obesity and other hyperalimentation | 47 (0.44) | Fang-feng-tong-sheng-san | 47 (0.01) | *Rheum officinale* Baill., radix and rhizome (*Da Huang*) | 47 (0.01) |
| 438 Late effects of cerebrovascular disease | 715 Osteoarthrosis and allied disorders | 564 Functional digestive disorders, not elsewhere classified | 43 (0.4) | Ba-wei-di-huang-wan | 19 (0.01) | *Aconitum carmichaeli* Debx., radix (*Pao Fu Zi*) | 9 (0.00) |
| 250 Diabetes mellitus | 401 Essential hypertension | 272 Disorders of lipoid metabolism | 39 (0.36) | Tian-ma-gou-teng-yin | 17 (0.00) | *Astragalus membranaceus* (Fisch.) Bunge., radix (*Huang Qi*) | 21 (0.01) |
| 716 Other and unspecified arthropathies | 401 Essential hypertension | 788 Symptoms involving urinary system | 38 (0.36) | Gui-lu-er-xian-jiao | 38 (0.01) | *Achyranthes bidentate* Blume., radix (*Hui Niu Xi*) | 38 (0.01) |
| 307 Special symptoms or syndromes, not elsewhere classified | 571 Chronic liver disease and cirrhosis | 571 Chronic liver disease and cirrhosis | 34 (0.32) | Suan-zao-ren-tang | 34 (0.01) | *Nelumbo nucifera* Gaertn., rhizome node (*Ou Jie*) | 34 (0.01) |
| 571 Chronic liver disease and cirrhosis | 574 Cholelithiasis | 710 Diffuse diseases of connective tissue | 30 (0.28) | Yin-chen-wu-ling-san | 30 (0.01) | *Scrophularia ningpoensis* Hemsl., radix (*Xuan Shen*) | 30 (0.01) |
| 414 Other forms of chronic ischemic heart disease | 272 Disorders of lipoid metabolism | 582 Chronic glomerulonephritis | 29 (0.27) | Liu-wei-di-huang-wan | 14 (0.00) | *Salvia miltiorrhiza* Bunge., radix (*Dan Shen*) | 19 (0.01) |
| 427 Cardiac dysrhythmias | 287 Purpura and other hemorrhagic conditions | 287 Purpura and other hemorrhagic conditions | 28 (0.26) | Chai-hu-jia-long-gu-mu-li-tang | 26 (0.01) | - | - |
